# Supplementary material for: A framework for the emergence and analysis of language in social learning agents
Source: Nat Commun. 2024 Aug 31;15:7590. doi: 10.1038/s41467-024-51887-5 (PMC11366021; doi:10.1038/s41467-024-51887-5)
Supplement: Supplementary file 1 — Supplementary Information [file 41467_2024_51887_MOESM1_ESM.pdf]

Supplementary Information

A framework for the emergence and analysis of language in social learning agents

Tobias J. Wieczorek[1,2], Tatjana Tchumatchenko[2], Carlos Wert-Carvajal[2,*,#], and Maximilian F. Eggl[2,†,#]

[1]Department of Computer Science, Technical University Darmstadt, Darmstadt, Germany
[2]Institute of Experimental Epileptology and Cognition Research, University of Bonn Medical Center, Bonn, Germany
[#] These authors jointly supervised this work.
[*]cwer1@uni-bonn.de, [†]meggl@uni-bonn.de

The effect of linearity in the autoencoder and the student

All our networks in our results were implemented with non-linear activation functions, so for completeness' sake we include the results arising from removing those non-linearities. The results of having linear student and autoencoder architectures can be seen in Supplementary Fig. S1, while a non-linear student and linear autoencoder is shown in Supplementary Fig. S2.

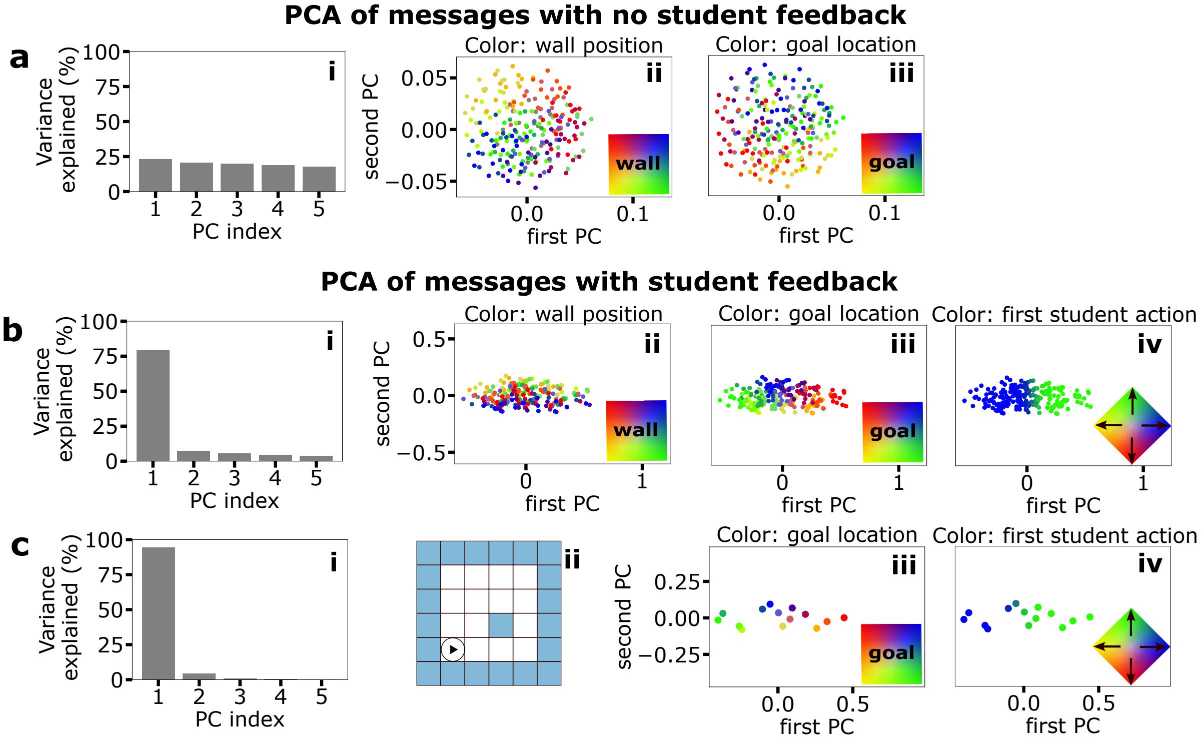

Figure S1. Removing the non-linearities of the autoencoder and student networks leads to a significantly altered embedding structure. a) Principal Component Analysis (PCA) of the lower-dimensional messages of size ($K = 5$) obtained from a language encoding without student feedback (eq. (4)) for all possible tasks in the 4×4 mazes with ≤ 1 walls. *i)* Explained variance by principal component. *ii)-iii)* depicts the messages highlighted by the position of the single wall (gray refers to the maze with no walls) and by the position of the goal, respectively. **b)** Result of the message encoding now including student feedback achieved by using eq. (1) for the loss function. *i)-iii)* depict the same concepts as in *a)*. *iv)* shows messages highlighted by preferred first student action (step up or right). **c)** PCA of the messages with student feedback from an example grid-world (depicted in *(ii)*).

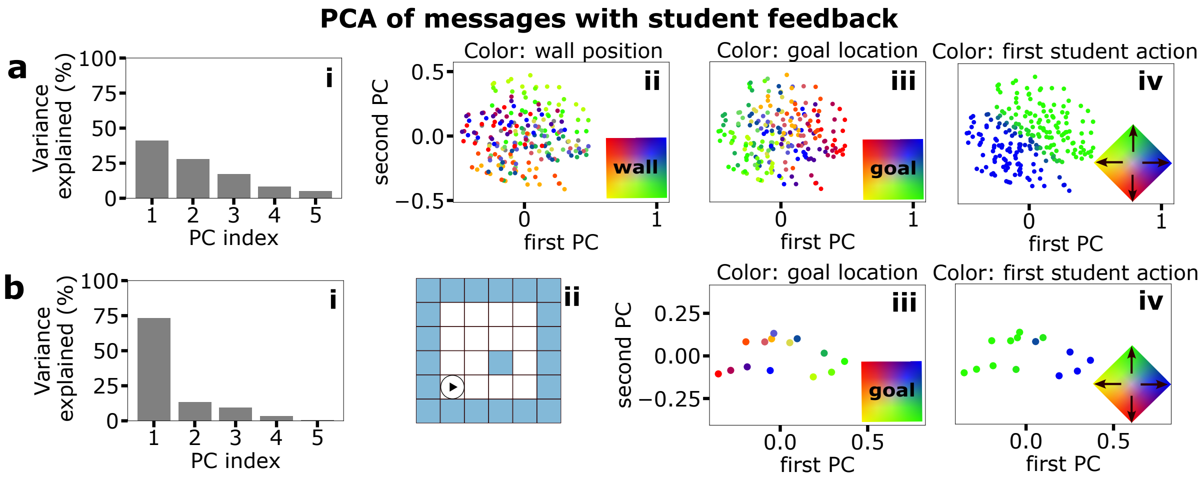

Figure S2. Reintroducing the non-linearity in the student and then applying student feedback, leads to an approximation of the structure of the full non-linear setup. **a)** Principal Component Analysis (PCA) of the lower-dimensional messages of size ($K = 5$) obtained from a language encoding with student feedback (eq. (4)) for all possible tasks in the 4×4 mazes with ≤ 1 walls. *i)* Explained variance by principal component. *ii)-iv)* depicts the messages highlighted by the position of the single wall (gray refers to the maze with no walls), by the position of the goal, and by preferred first student action (step up or right). **b)** PCA of the messages with student feedback from an example grid-world (depicted in *(ii)*).

Student trained on “frozen“ messages

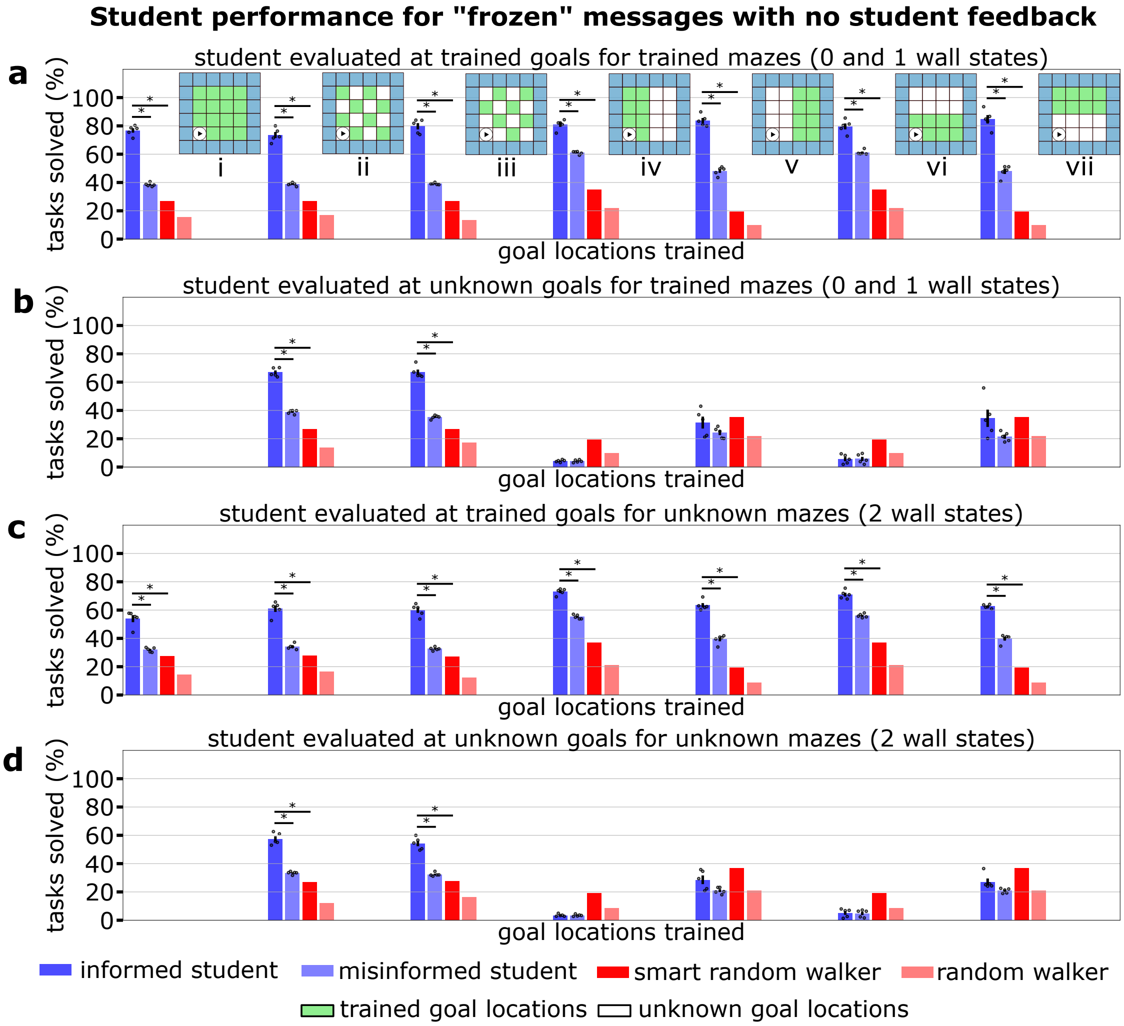

Figure S3. Training a student on a set of frozen lower-dimensional representations, arising from an independently trained language (without feedback), leads to reasonable task performance. However, this performance is worse than the student-feedback version (cf. Fig. 4). a)-d) A student is trained on a set of frozen messages arising from an independently trained language (created without feedback) and then compared against a misinformed student and two random walkers. The comparison is once more performed for the seven sets of trained goal locations, *(i)-(vii)*. The relevant tasks per panel are identical to Fig. 4. $*$ refers to $p < 0.05$ where the p is obtained using a two-sided and one-sided t-test (multiple tests were accounted for using a Bonferroni correction factor) for the informed vs misinformed and smart random walker, respectively. In all of the above, bars represent the mean task performance $\pm$ the SEM.

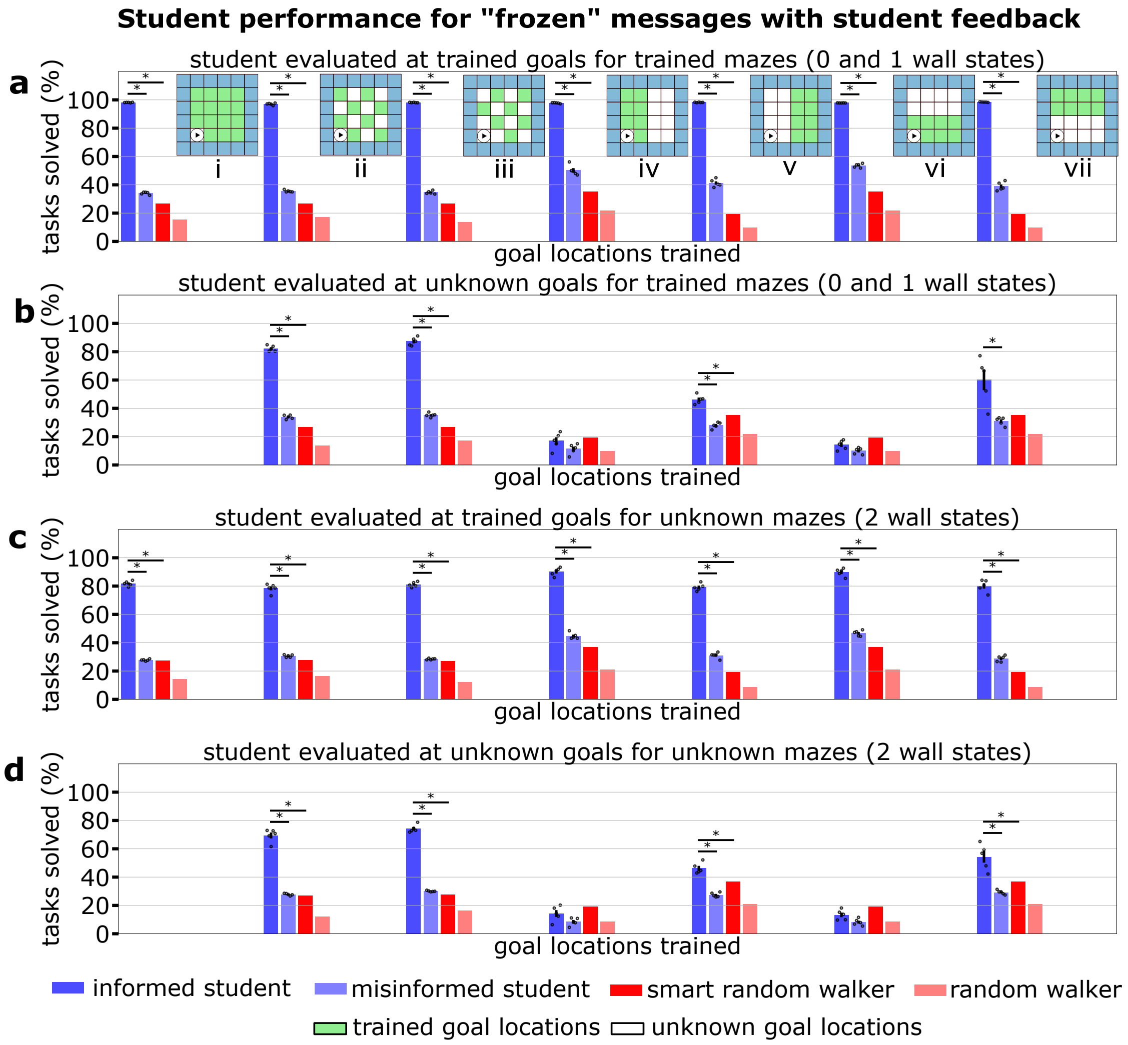

Figure S4. Training a student on set of frozen lower-dimensional representations arising from an independently trained language (with feedback), leads to better performance than when the language is allowed to change mid-training (cf. Fig. 4). a)-d) A student is trained on a set of frozen messages arising from an independently trained language (created with feedback) and then compared against a misinformed student and two random walkers. The comparison is once more performed for the seven sets of trained goal locations, *(i)-(vii)*. The relevant tasks per panel are identical to Fig. 4. * refers to $p < 0.05$ where the p is obtained using a two-sided and one-sided t-test (multiple tests were accounted for using a Bonferroni correction factor) for the informed vs misinformed and smart random walker, respectively. In all of the above, bars represent the mean task performance $\pm$ the SEM.

Teacher and student Q-matrices

We also performed PCA directly on the teacher and student Q-matrices, to see if the structures and features that emerged were comparable to the language encoding.

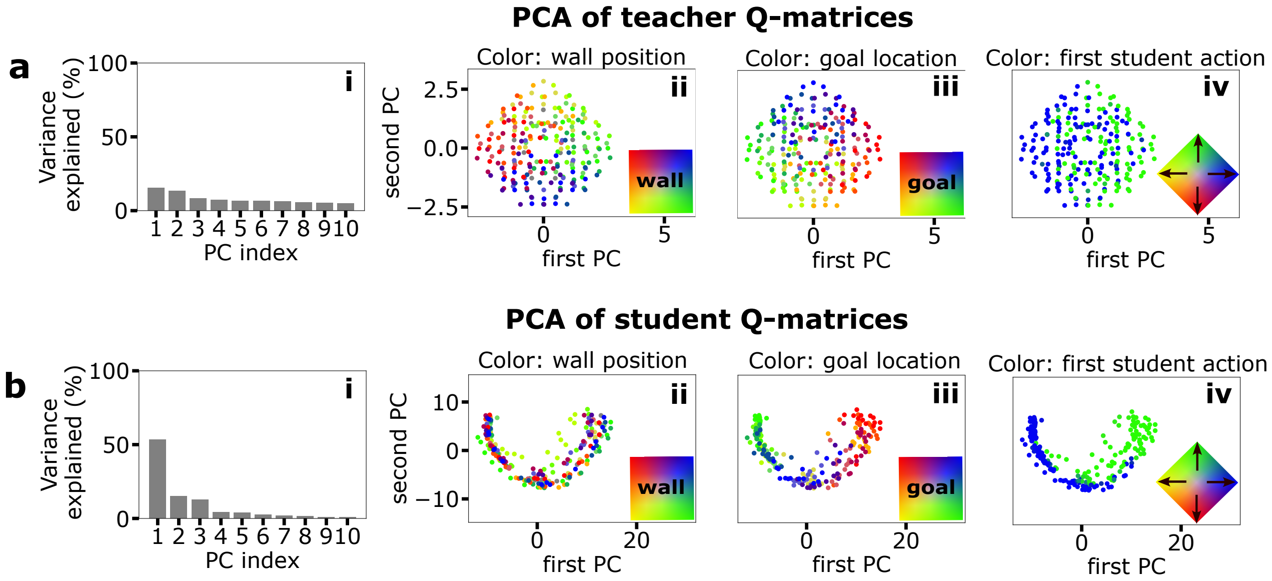

Figure S5. PCA applied directly to the task information from the student and teachers reveals structure that mirrors the tasks themselves. a) PCA of the Q-matrices learned by the teacher networks. Each data point corresponds to a Q-matrix and, therefore, to a maze task. From left to right the figures show *(i)* the variance explained by the first 10 PCs, and projections of the matrices to the first two PCs with coloration by *(ii)* maze identity, *(iii)* goal location and *(iv)* initial student action. **b)** PCA of the student "Q-matrices", which are task information matrices learned by the student, but correspond to action probabilities instead of correct Q-values. The subfigures are identical to (a). The data dimensionality in both cases is $\tilde{n} \times \tilde{n} \times 4$ $(= 64$ for maze size $\tilde{n} = 4)$.

**Message grouping**	$\mathbf{Var_{within}}(X)$	$\mathbf{Var_{between}}(X)$	β	F-value
By wall position (Supplementary Fig. S5a(ii))	552	1895	0.774	47.81
By goal location (Supplementary Fig. S5a(iii))	1829	618	0.253	4.71
By wall position (Supplementary Fig. S5b(ii))	23235	5147	0.181	3.09
By goal location (Supplementary Fig. S5b(iii))	9270	19113	0.673	28.72

Table S1. Analysis of variance for world groups and goal groups in the matrix spaces from Supplementary Fig. S5 according to eq. (6) - eq. (11). The statistical test performed was an one-sided F-test for variance analysis of groups.

Dimensionality reduction methods

To gain a deeper understanding of the hidden representations (see, for example, the limitations of PCA analysis[80]) we perform linear discriminant analysis (LDA) as well as two different non-linear dimensionality reduction methods on the messages from a language created without feedback and one created with feedback (the same as in Fig. 2 in the main results). Both non-linear methods, t-SNE[81] and UMAP[82], come with tunable parameters, the most crucial of which are the perplexity π (t-SNE) and the number of neighbors k (UMAP), which can be seen as the number of nearest neighbors that are taken into account for each data point when designing the low-dimensional representation of the data. In the following, the subscript "all" on these parameters refers to the case of messages from all trained worlds, whereas the subscript "single" refers to the case of messages from only a single world. We experiment with comparatively (with respect to the number of samples) low (Supplementary Fig. S6, Fig. S8) and comparatively high (Fig. S7, Supplementary Fig. S9) values for π and k, respectively. All other parameters, namely the number of iterations in t-SNE ($= 1500$) and the minimum distance between data points in UMAP ($= 0.5$), are kept constant.

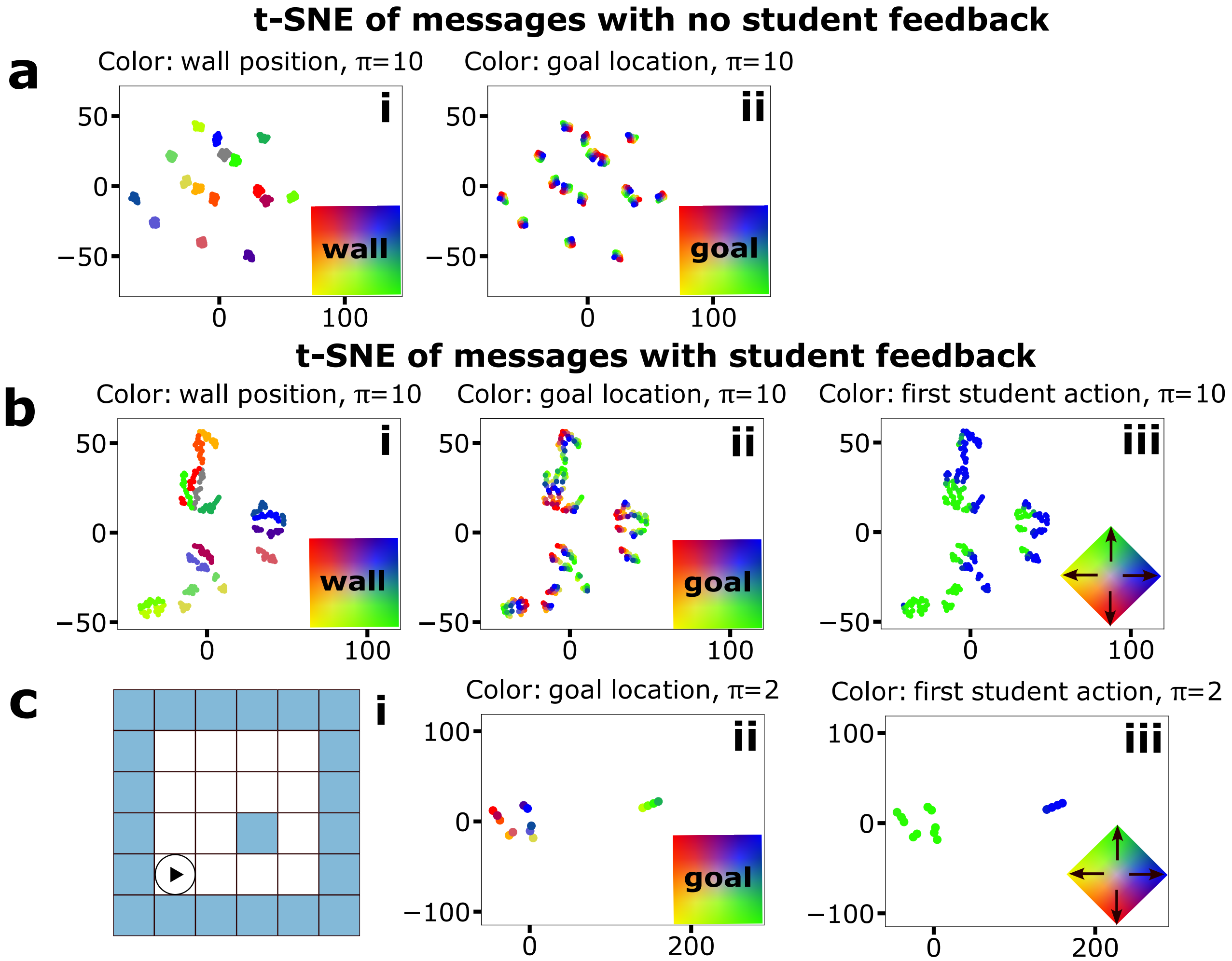

Figure S6. t-SNE of messages, low perplexity parameter. We apply the t-SNE dimensionality reduction method (perplexities $\pi_{\text{all}} = 10$ and $\pi_{\text{single}} = 2$) to both the language created without student feedback and the one created with student feedback from Fig. 2 in the main results. The individual panels are conceptually identical to Fig. 2: **a)** shows all messages from the language created without feedback, **b)** all messages from the language created with feedback and **c)** those messages from the language analyzed in *b)*, which refer to tasks in the maze shown in *c) i*. The coloration of the messages is done by wall position, goal location of the task and (in the cases of feedback) the probability of the first student action, respectively.

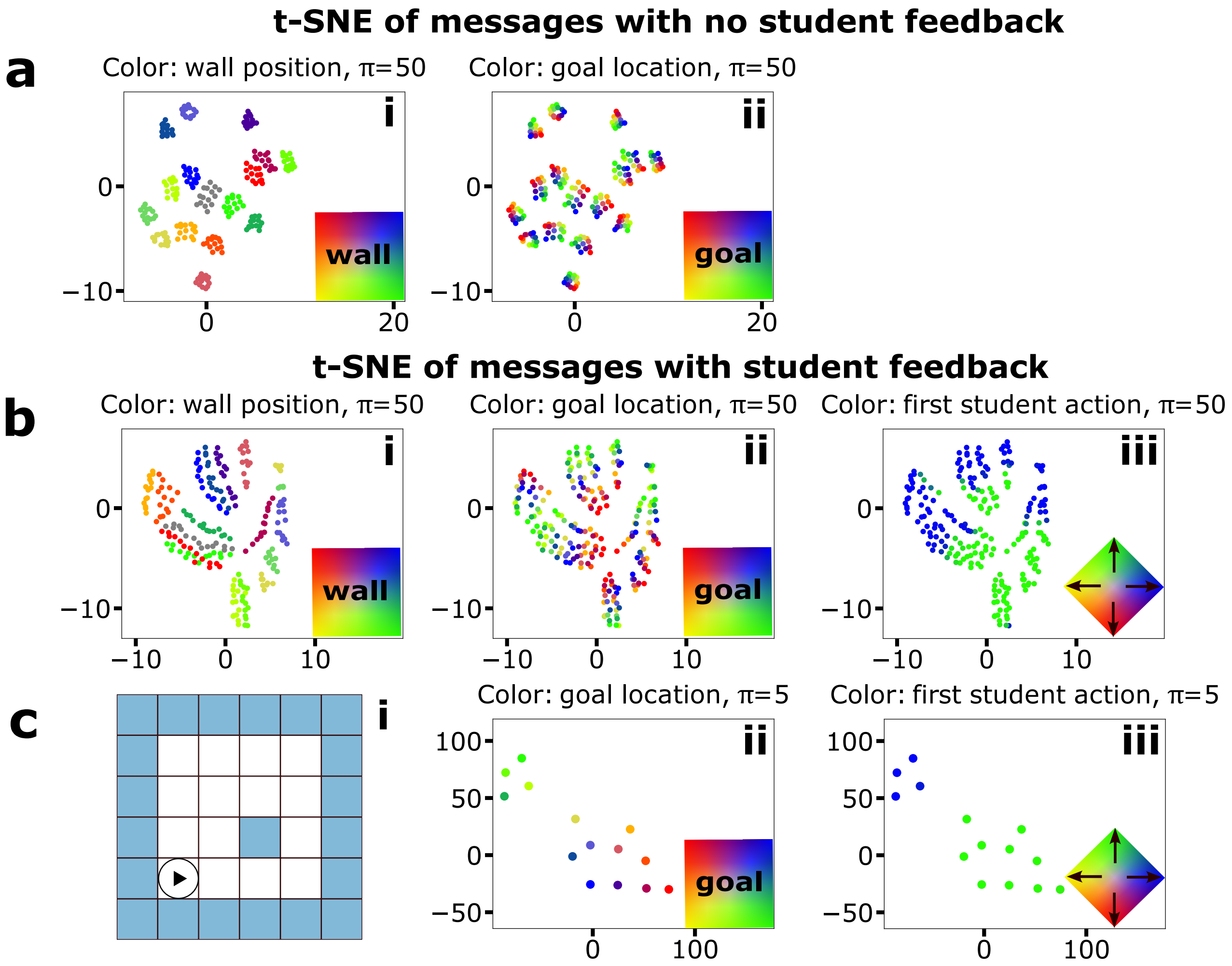

Figure S7. t-SNE of messages, high perplexity parameter. We apply the t-SNE dimensionality reduction method (perplexities $\pi_{\text{all}} = 50$ and $\pi_{\text{single}} = 5$) to both the language created without student feedback and the one created with student feedback from Fig. 2 in the main results. The individual panels are conceptually identical to Fig. 2: **a)** shows all messages from the language created without feedback, **b)** all messages from the language created with feedback and **c)** those messages from the language analyzed in *b)*, which refer to tasks in the maze shown in *c) i*. The coloration of the messages is done by wall position, goal location of the task and (in the cases of feedback) the probability of the first student action, respectively.

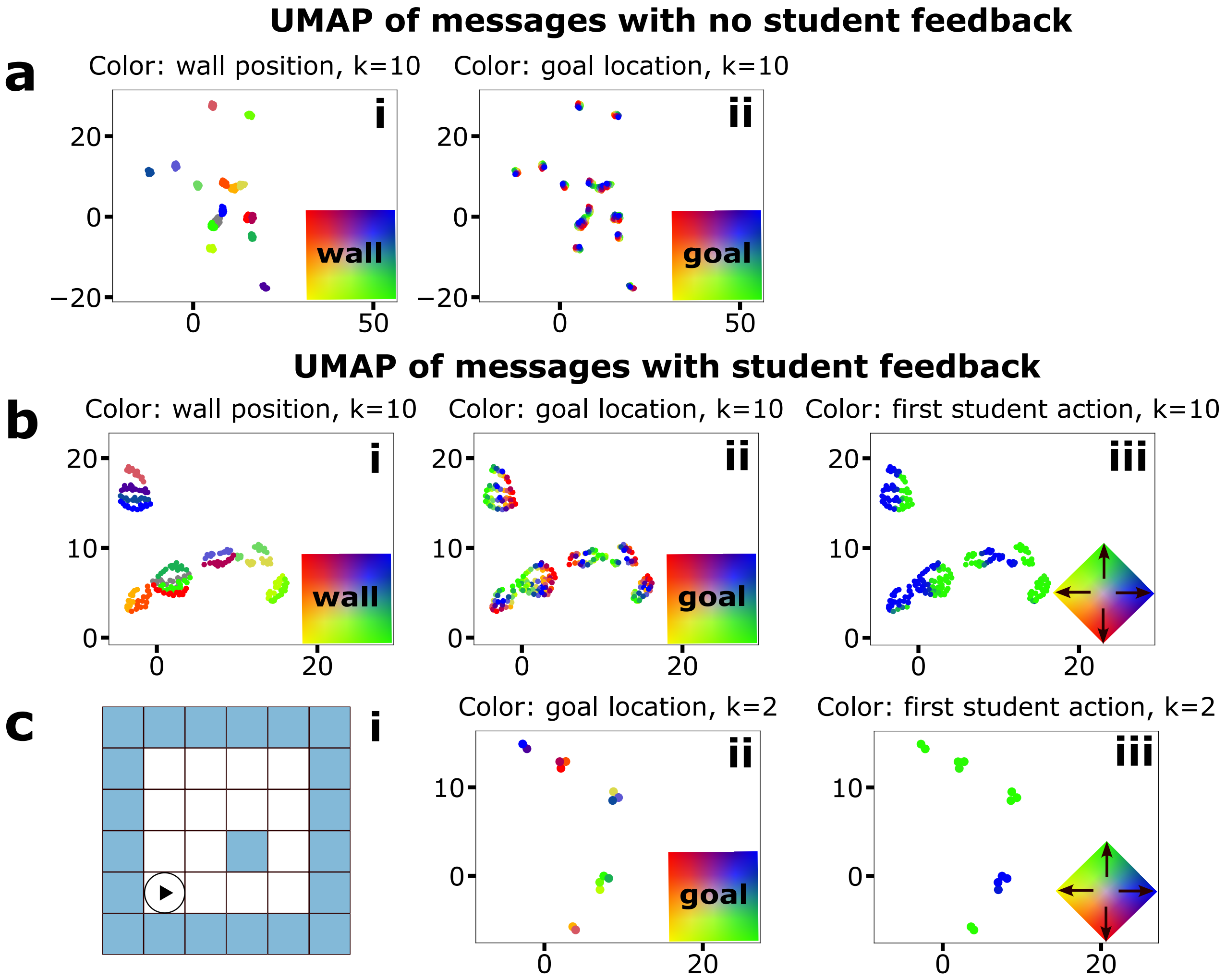

Figure S8. UMAP of messages, low neighbors parameter. We apply the UMAP dimensionality reduction method (neighbors $k_{\text{all}} = 10$ and $k_{\text{single}} = 2$) to both the language created without student feedback and the one created with student feedback from Fig. 2 in the main results. The individual panels are conceptually identical to Fig. 2: **a)** shows all messages from the language created without feedback, **b)** all messages from the language created with feedback and **c)** those messages from the language analyzed in *b)*, which refer to tasks in the maze shown in *c) i*. The coloration of the messages is done by wall position, goal location of the task and (in the cases of feedback) the probability of the first student action, respectively.

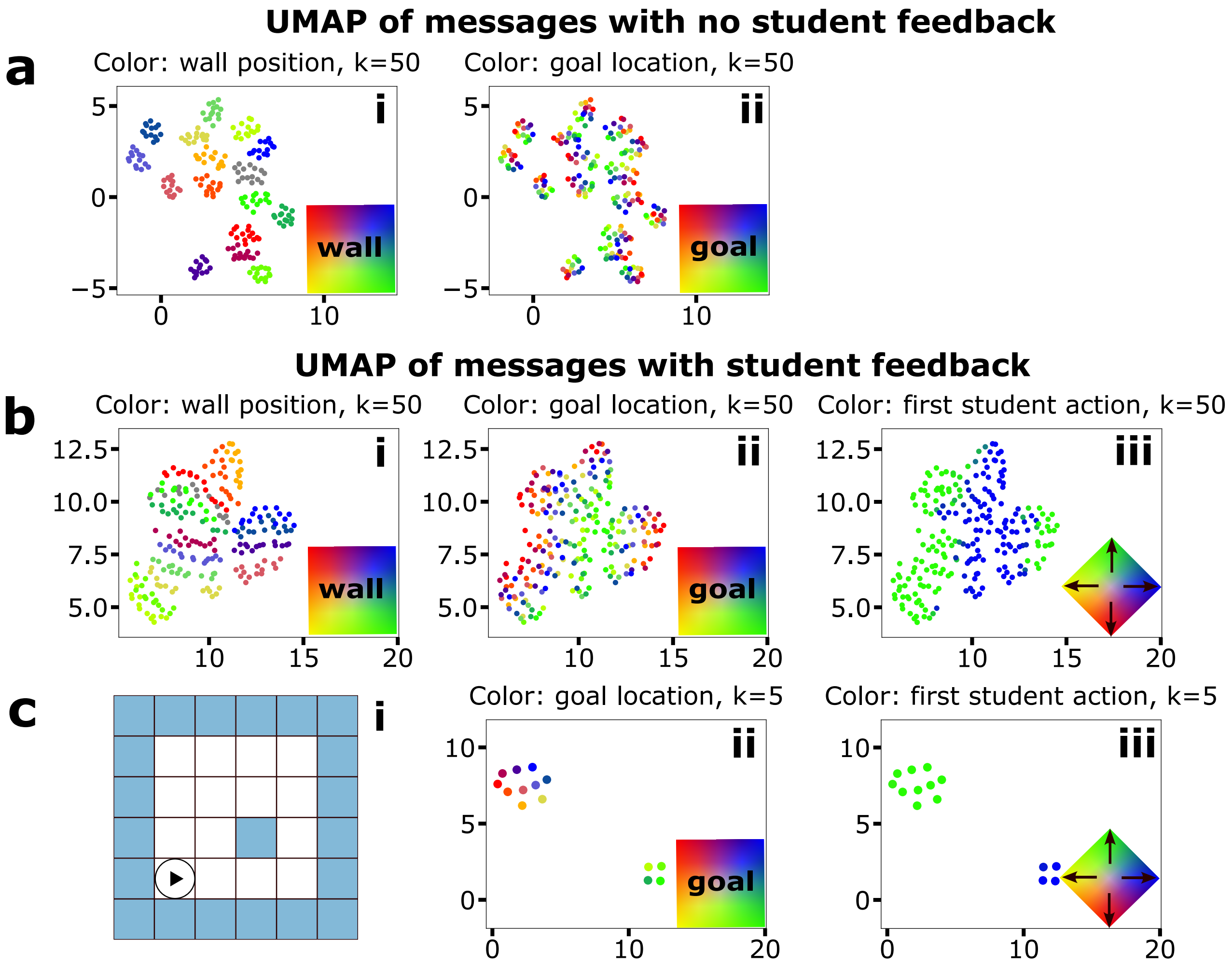

Figure S9. UMAP of messages, high neighbors parameter. We apply the UMAP dimensionality reduction method (neighbors $k_{\text{all}} = 50$ and $k_{\text{single}} = 5$) to both the language created without student feedback and the one created with student feedback from Fig. 2 in the main results. The individual panels are conceptually identical to Fig. 2: **a)** shows all messages from the language created without feedback, **b)** all messages from the language created with feedback and **c)** those messages from the language analyzed in *b)*, which refer to tasks in the maze shown in *c) i*. The coloration of the messages is done by wall position, goal location of the task and (in the cases of feedback) the probability of the first student action, respectively.

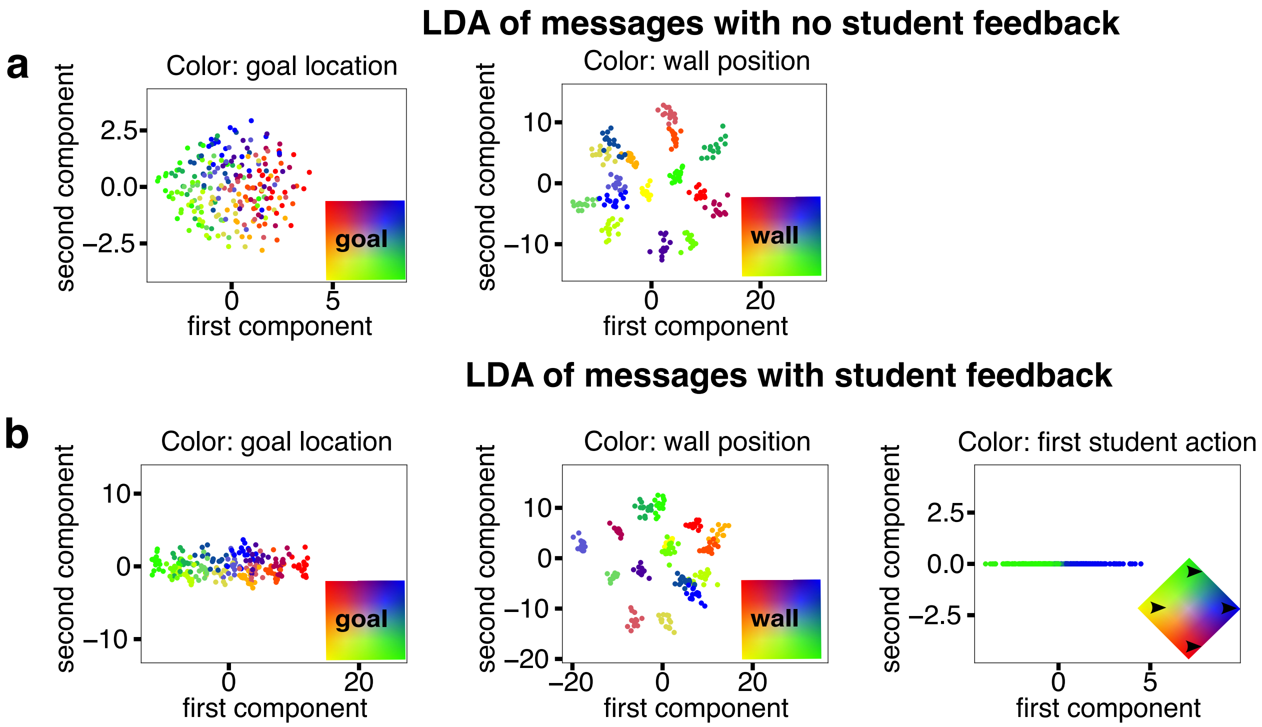

Figure S10. LDA projection of messages. Messages projected over the two main components obtained from LDA for each label, following Fig. 2. The individual panels are conceptually identical to Fig. 2: **a)** shows all messages from the language created without feedback, and **b)** all messages from the language created with feedback. Wall label accuracy is 100% without students, 99.6% with feedback. Goal location is less accurate, at 28.9% without students and 75.6% with feedback.

Autoencoder loss plots for different hyperparameters

The robustness of the results from Fig. 4a-d was checked by varying the hyperparameter ζ, which controls the relative importance of the autoencoder (SAE) and student goal finding losses (eq. (1)). The results in the main text (Fig. 4a-d) were obtained with $\zeta = 5$, but Supplementary Fig. S11a(iv)-d(iv) show that the results hold for $\zeta \in \{1, 2, 10\}$ as well.

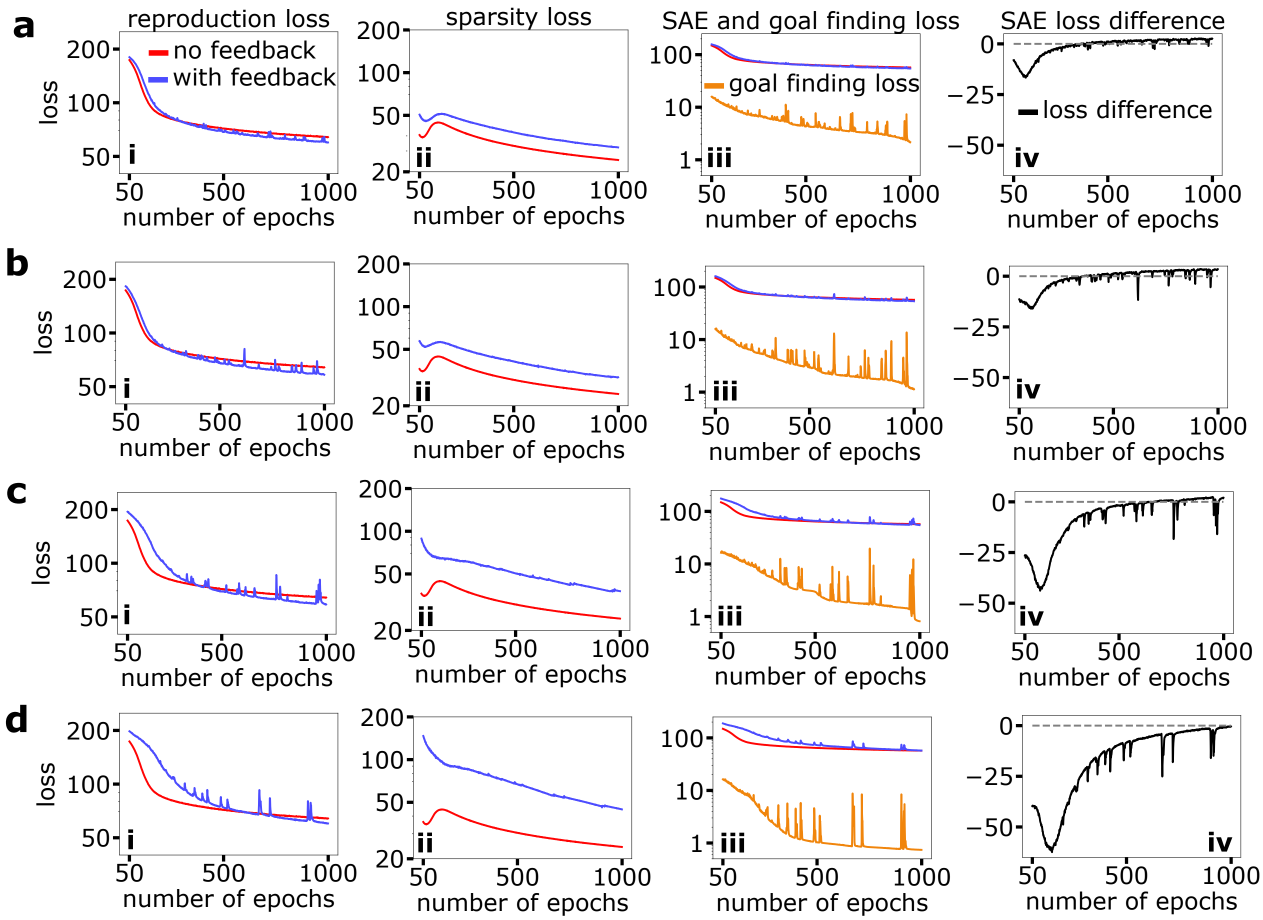

Figure S11. The compound autoencoder with student feedback loss is lower for a range of different values of the hyperparameter ζ (the student-feedback weighting). Each row (**a)-d)**) represents a different value for $\zeta = 1, 2, 5, 10$. From left to right the plots show (i) $\mathscr{L}_{\text{reconstruction}}$ and $\mathscr{L}_{\text{reconstruction, feedback}}$, (ii) $\mathscr{L}_{\text{sparsity}}$ and $\mathscr{L}_{\text{sparsity, feedback}}$, (iii) $\mathscr{L}_{\text{SAE}}$, $\mathscr{L}_{\text{SAE, feedback}}$ and $\mathscr{L}_{\text{goal finding}}$, (iv) $\mathscr{L}_{\text{SAE}} - \mathscr{L}_{\text{SAE, feedback}}$. For reference see eq. (1) and eq. (4).

Hyperparameters
In table S2, we list the hyperparameters used in our model and their values for different simulations. As we aimed to study the emergent language structure rather than achieve the best performance, we avoided performing a costly hyperparameter search. Instead, values were chosen that lead to reasonable training times and performance. Example simulations have given us good reason to expect stability of the major findings across values of the message length K, the learning rate α_{Adam} and also γ and κ, which relate to the student loss function.

We separate the set of hyperparameters into two groups: task setup and teacher learning (upper half) and language training and student evaluation (lower half). The hyperparameters in the upper half have no significant impact on the communication protocol developed as they are relevant only for teacher learning of the navigation task.

The parameters chosen for this work represent a stable regime that allowed us computational tractability while still observing interesting features. Given the stability observed in the hyperparameters for the key features of this work (such as the effect of student feedback on the auto-encoder performance, see figures S6 and S8), we note that changes to these parameters would not have led to significantly different conclusions. Nonetheless, no formal hyperparameter optimization was done, as the purpose of this work was to study the possibilities of our proposed framework, such as the structures of the message space, which we observed were largely independent of the hyperparameters.

**Hyperparameter**	**usage/meaning**	**value**	**figures used**
n	grid-world dimension (including outside walls)	6	all
$\tilde{n}$	grid-world dimension (without outside walls)	4	all
γ_{Bellman}	temporal discount in teacher Q-learning	0.99	all
R_{goal}	goal reward in teacher Q-learning	2	all
R_{wall}	wall reward in teacher Q-learning	-0.5	all
R_{step}	step reward in teacher Q-learning	-0.1	all
L	short-term memory size in teacher Q-learning	50	all
K	message length	5	all
α_{Adam}	learning rate in language training	5×10^{-4}	all
N_{epochs}	epoch number in language training	1000	all
γ	language training loss weighting	$\frac{1}{20}\sqrt{\frac{4\tilde{n}^2}{K}}$	all
ζ	language training loss weighting	5<br>1,2,5,10	all except Supplementary Fig. S11<br>Supplementary Fig. S11
κ	language training loss weighting	$\frac{1}{500}$	all
s	allowed steps per task in student evaluation	$2s_{\text{opt}}$	all

Table S2. The hyperparameters we used in our model with a brief description as to their usage and their values in the simulations used for creating the different figures. The parameters can roughly be separated into two blocks - the upper block are parameters for grid-world creation and teacher learning of the navigation tasks, whereas the lower block contains parameters for language creation and student evaluation.

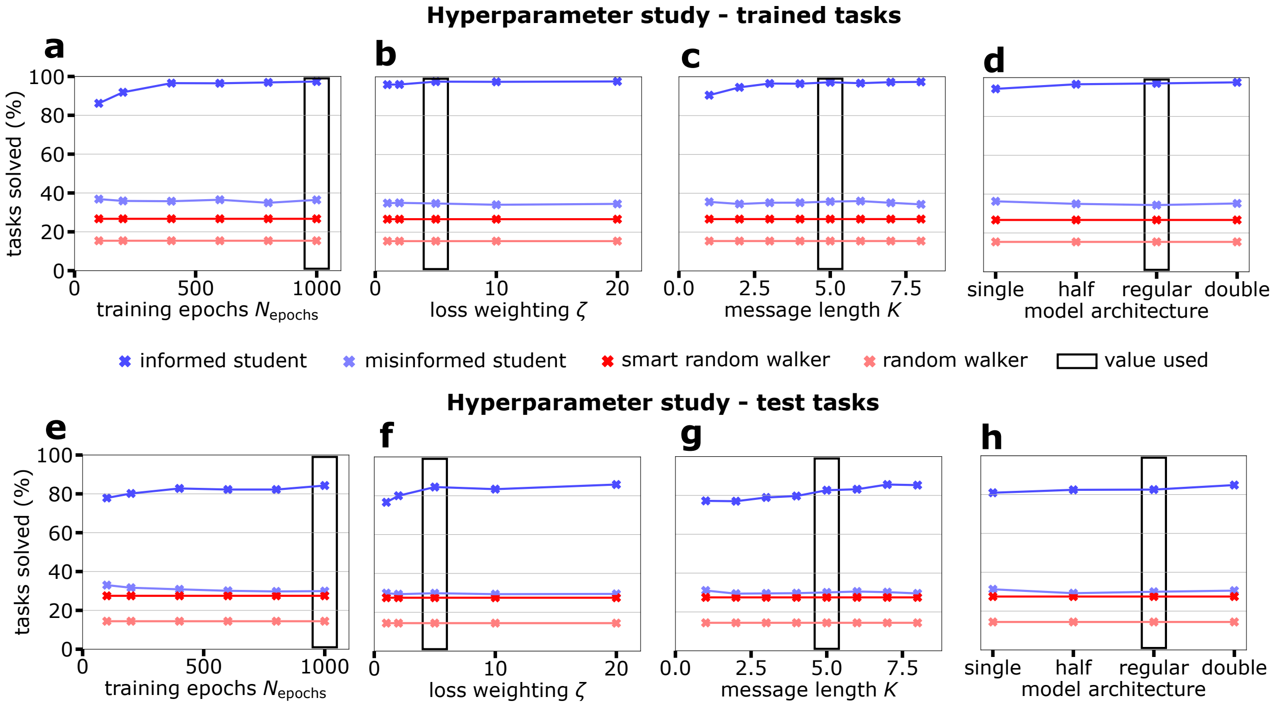

Figure S12. Effect of varying the key parameters of our communication protocol on the student performance. We tested the effect of varying several hyperparameters on the student performance on the trained tasks (*a)-d)*, all tasks in 4×4 mazes with 0 or 1 wall state) and on the test tasks (*e)-h)*, all tasks in 4×4 mazes with 2 wall states). The four hyperparameters we judged to have the most qualitative impact on the communication protocol were varied, namely the number of training epochs in *a), e)*, the loss weighting ζ in *b), f)* - see eq. (1) -, the length of the messages in *c), g)* and finally the student network's architecture in *d), h)*. The "regular" architecture is the one described in table 3, half/double refers to networks where number of neurons in all hidden layers are halved/doubled, respectively. The "single" defines a network where the three hidden layers of the student are replaced by just one hidden layer with 10 neurons. The hyperparameter values we consistently chose for all our main results are highlighted - see table S2 for reference. We note that in the majority of cases, the parameters chosen for our study (black boxes) represent a stable regime.

Closing the loop: Effect on student performance if all languages are included

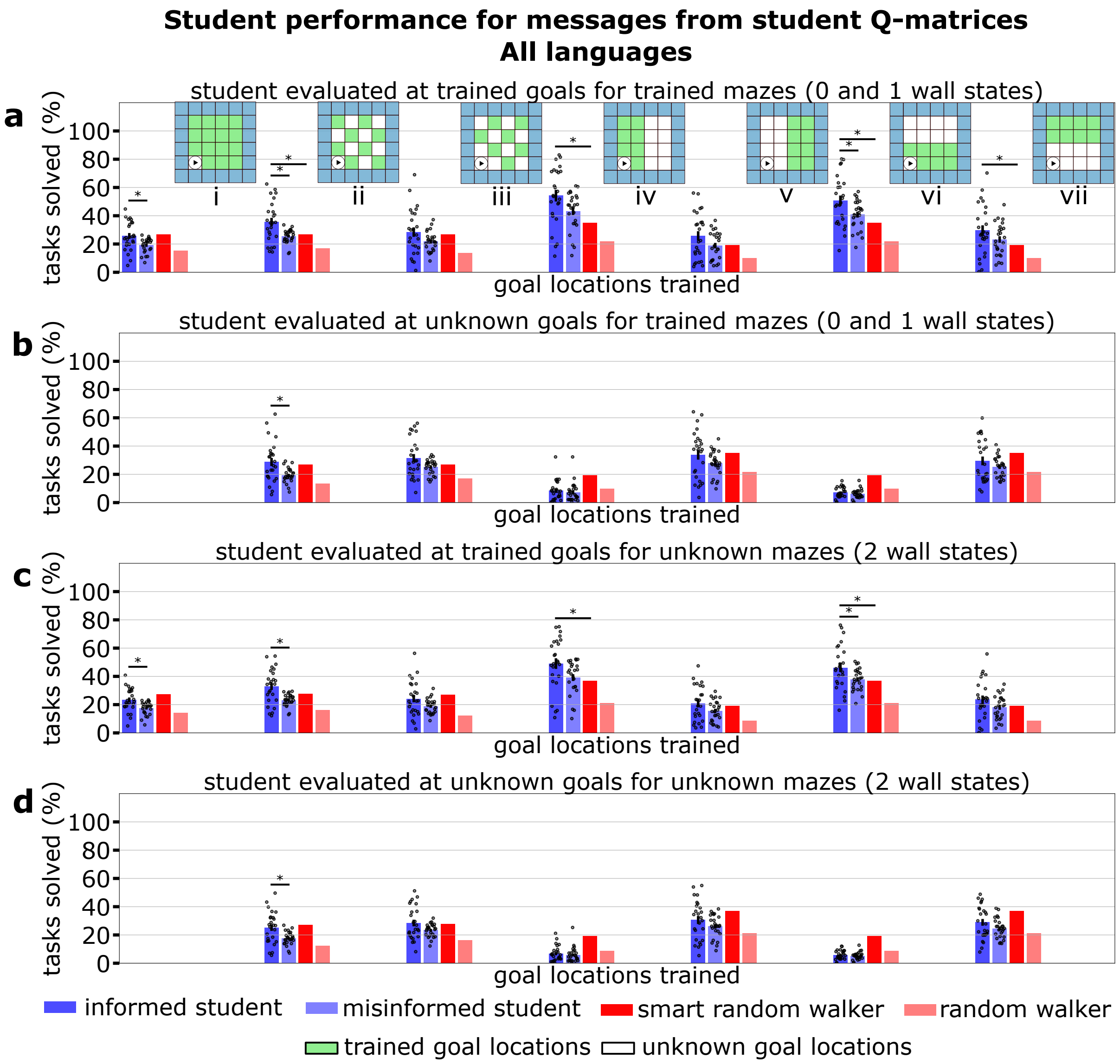

Figure S13. When including all languages in the student message generation, a lower informed student performance is observed in comparison to when only beneficial languages are retained (cf. Fig. 5). Nonetheless, the informed student still performs better than the random/misinformed student at the majority of the tasks. a)-d) Informed student performance on training and test maze tasks (see Methods for details) is compared against the misinformed student and two random walkers. The comparison is once more performed for the seven sets of trained goal locations, *(i)-(vii)*. The relevant tasks per panel are identical to Fig. 5. * refers to $p < 0.05$ where the p is obtained using a two-sided and one-sided t-test (multiple tests were accounted for using a Bonferroni correction factor) for the informed vs misinformed and smart random walker, respectively. In all of the above, bars represent the mean task performance $\pm$ the SEM.
